# Infant Methemoglobinemia: Causative Factors

**DOI:** 10.1289/ehp.113-a805b

**Published:** 2005-12

**Authors:** Catherine Zeman

**Affiliations:** Health Division, University of Northern Iowa Cedar Falls, Iowa, E-mail: catherine.zeman@uni.edu

That individually and environmentally mediated cofactors function in the development of infant methemoglobinemia (iMHG) is not a new finding. Studies cited by Fewtrell (2004) note these cofactors. In my work on iMHG, using a nested case–control study that was not cited by Fewtrell (2004), I confirmed that cofactors (feeding practices, individual and infant physiology, etc.) played a role in the disease status of populations under study (Zeman 2000; Zeman et al. 2002a).

Cofactor work completed with Ustyogova et al. (2002) indicated that *in vitro* studies examining exposures below and above the maximum contaminant limit for nitrate show impacts to lymphocyte proliferation and cytokine production with shift in immune response from a Th 1 lymphocyte immune status to a Th 2 lymphocyte, indicating possible decreased resistance to pathological states. Could this be another factor in iMHG? Ustyogova et al. (2002) examined healthy adults, but the study raises the issue of the effects of exposure on the developing immune system of infants. The microbial status of drinking water for participants in the case–control study (Bauer et al. 2003), has been evaluated at the bacterial and parasite levels (Bauer et al. 2003, Zeman et al. 2005). Findings indicated that most water was highly contaminated with fecal coliforms (0–1,000/100 mL) and protozoan oocysts (0–84 cysts/L); when the likelihood of contamination was compared to data on whether or not an iMHG case had occurred in the household, no significant relationship was found.

Fewtrell (2004) claimed that no exposure–response data are available, but two articles (Zeman et al. 2002a, 2002b) reporting on the iMHG case–control study and associated exposure assessment to nitrate/nitrite contradict this. In one of these studies (Zeman et al. 2002a), a bivariate fit of nitrate level in well water and nitrite exposure through water and dietary sources (*p* = 0.0001) validated the exposure assessment methodology. Table 9 of this article illustrates the relationship strength under bivariate test for a variety of risk factors, and Table 11 provides a multivariate analysis showing the most predictive factors for this study population—exposure to drinking water nitrates, breast-feeding duration, and lack of vitamin use (Zeman et al. 2002a). By stratifying these data for bivariate analysis and comparing the calculated nitrite exposure for each child for low to medium (< 0.1 mg/kg/day to ≥0.1–1.5 mg/kg/day) and low to high (< 0.1 mg/kg/day to ≥1.5 mg/kg/day) exposures, the likelihood (*L*) and Pearson (P) calculations show a definite gradation in effect and significance in both situations: low to medium (*L* = 6.574, *p* = 0.0103; and *P* = 4.377, *p* = 0.0364); low to high (*L* = 20.7474, *p* = 0.0001; and *P* = 15.605, *p* = 0.0001). I agree, however, that no dose–response relationship has been documented comparing calculated exposure to measured blood methemoglobin level at the time of a clinically diagnosed iMHG case. This would be a gold standard that would help us to tease out the causative factors of iMHG and to establish solidly or refute what looks like, to date, the centrality of the role of nitrate exposure in the etiology of iMHG.
